# Experimental realization of visible gas sensing technology based on spatial heterodyne spectroscopy

**DOI:** 10.1038/s41598-022-05510-6

**Published:** 2022-01-26

**Authors:** Wen-li Zhang, Zhao-yu Liu, Kun Liang, Yi Wang, Ke-fan Chen, Yao-wei Sun, Sheng Wang

**Affiliations:** 1 Zhengzhou University of Aeronautics, Henan, 450002 China; 2 University of Electronic Science and Technology of China, Sihuan, 611731 China; 3 Henan Electric Power Hospital, Henan, 450052 China

**Keywords:** Environmental monitoring, Optical physics

## Abstract

Based on the characteristics of optical absorption gas sensing technology (OA-GST) and spatial heterodyne spectroscopy (SHS), a novel type of visual gas sensing technology (V-GST) can present the invisible gas information in the form of two-dimensional visual fingerprint, which has attracted people's attention. In this paper, we have realized the NO_2_ detection of V-GST in the laboratory environment for the first time. Experimental results show that: V-GST not only has different interferogram response to different spectra, but also has good response to different concentrations of NO_2_, which lays a foundation for the application of this technology in gas sensing. And the average classification recognition rate of the system for different band NO_2_ response data is over 80%, which verifies the effectiveness of the V-GST in gas detection.

## Introduction

Electronic nose (E-nose)^1,2^ based on the principle of bionics can provide more accurate and objective gas detection results, and has been widely used in food engineering^3,4^, healthcare^5,6^, environmental protection^7,8^, social security^9,10^, etc. However, the application prospect of the E-nose is affected because of some shortcomings of the E-nose gas sensing system, such as small sensor array, poor stability, low detection accuracy and longer response time^11,12^. As a commonly used gas sensing technology, optical absorption gas sensing technology (OA-GST)^13,14^ not only has a faster response time, but also provides a larger sensor array and a wider spectral response range^15^, which is very suitable to undertake the gas sensing task of the E-nose. Otherwise, as a non-contact detection method, it can detect high temperature, high humidity and corrosive gases. Hence, OA-GST has received considerable attention in E-nose.

As a core component in OA-GST, however, the existing spectrometers have some defects, which limit the application of OA-GST in E-nose. Such as, the grating spectrometer have the problem of mutual restriction between response spectral range and spectral resolution, the Fourier transform spectrometer have higher requirements for the stability of the application environment. Fortunately, as a new interferometric spectral detection technique, the spatial heterodyne spectroscopy (SHS)^16,17^ not only has the characteristics of strong stability, but also has ultra-high spectral resolution, which can realize gas detection with fine structure characteristic spectrum. Therefore, the application of OA-GST and SHS in E-nose has its unique advantages^18^. Firstly, the OA-GST can realize effective sensing of gas characteristic information. Secondly, SHS can present the spectrum with gas absorption spectrum in the form of two-dimensional (2D) interferogram. Thirdly, the ultra-high spectral resolution makes the spectral information of the gas detected by the system more detailed, which improves the accuracy of the system for gas detection. Furthermore, the interferogram of the SHS is dependent on the wavenumber distribution of the input light source completely, which allows us to make a simple judgment of the input spectral information by the structure of the interferogram. Therefore, the visual gas sensing technology (V-GST) that combines OA-GST and SHS has attracted people's attention for its novel gas detection method. Unfortunately, this program is only in the stage of theoretical analysis^18^, and has not yet realized the effective detection of gas in the laboratory environment.

In order to verify the effectiveness of V-GST, firstly, this paper explains the theoretical model of V-GST and analyzes the feasibility of this model for gas sensing. Then a dual excitation light source is used to build the V-GST experiment platform, and analyzed the effectiveness of the gas sensing system. Finally, the wide spectrum light source is used to collect the response transmittance maps of NO_2_ in different bands, and the image feature extraction algorithm, pattern recognition algorithm are used to analyze the response data.

## Theoretical model of visual gas sensing technology

The novel visual gas sensing mechanism is combining the basic principles of OA-GST^15^ with wide spectral spatial heterodyne spectrometer (WS-SHS)^19,20^. Among them, OA-GST is used to obtain the 1D sensing spectra of the test gas. And from Lambert–Beer Law^21^, the absorption spectrum of the test gas can be expressed as follows

1 $$ B_{{{\text{out}}}} (\sigma ) = B_{{{\text{in}}}} (\sigma )e^{ - \alpha (\sigma )CL} $$

where $\sigma$ is the characteristic wavenumber of the test gas, $B_{{{\text{in}}}} (\sigma )$ is the input spectrum, $B_{{{\text{out}}}} (\sigma )$ is the absorption spectrum, $\alpha (\sigma )$ is the absorption coefficient which reflects the characteristics of gas, $C$ is the concentration of the test gas, $L$ is the effective optical path.

Another core component WS-SHS is used to present the sensing spectrum of test gas into 2D response interferogram. Based on the basic theory of WS-SHS^19,20^, the corresponding 2D response interferogram of the input spectrum and the absorption spectrum are expressed as^18^

2 $$ I_{{{\text{in}}}} (x,y) = \sum\limits_{m} {\int_{0}^{\infty } {B_{{{\text{in}}}} (\sigma )F_{m} (\sigma )(1 + \cos (2\pi (f_{x} \cdot x + f_{y} \cdot y)))d\sigma } } $$

3 $$ I_{{{\text{out}}}} (x,y) = \sum\limits_{m} {\int_{0}^{\infty } {B_{{{\text{in}}}} (\sigma )e^{ - \alpha (\sigma )CL} F_{m} (\sigma )(1 + \cos (2\pi (f_{x} \cdot x + f_{y} \cdot y)))d\sigma } } $$

where $m$ is the diffractive order of the Echelle grating, $F_{m} (\sigma )$ is the diffractive efficiency of grating for order $m$, $x$ and $y$ are the pixel distributions of the interferogram in the horizontal and vertical directions, $f_{x} = 4(\sigma - \sigma_{0m} )\tan {\uptheta }$ and $f_{y} = \alpha \sigma$ are represents the spatial frequency of the x-axis and y-axis, $\sigma_{0m}$ is the Littrow wavenumber for order $m$ of the Echelle grating.

According Eqs. (2) and (3), define a transmittance map of the test gas as

4 $$ T(x,y) = \frac{{I_{{{\text{out}}}} (x,y)}}{{I_{{{\text{in}}}} (x,y)}} = \frac{{\sum\limits_{m} {\int_{0}^{\infty } {G(\sigma ,x,y)e^{ - \alpha (\sigma )CL} d\sigma } } }}{{\sum\limits_{m} {\int_{0}^{\infty } {G(\sigma ,x,y)d\sigma } } }} $$

where $G(\sigma ,x,y) = B_{{{\text{in}}}} (\sigma )F_{m} (\sigma )(1 + \cos (2\pi (f_{x} \cdot x + f_{y} \cdot y)))$. From Eq. (4), it can be found that: for a fixed system, when the test gas has the same concentration ($C$ is same) but with the different type ($\alpha (\sigma )$ is different), the difference of the transmittance map ($T(x,y)$ is shown in the structure and period of interference fringes; when the test gas has the same type ($\alpha (\sigma )$ is same) but with the different concentration ($C$ is different), the difference of the transmittance map ($T(x,y)$) is shown in the brightness of interference fringes. According to the theoretical basis of spectral analysis technology, the above principle can be used as the gas sensing mechanism of the V-GST.

## Experimental platform of visual gas sensing technology

According to the theoretical model of the V-GST (shown in Eq. (4)), we can find that with the increase of the diffraction order of Echelle gratings, the spectral detection range of the system can traverse the ultraviolet–visible-infrared band. While, in order to build a stable and effective V-GST experimental platform, a dual excitation light source (the light source emits two kinds of narrowband light with different center wavelengths at the same time) is used for the calibration of the experimental platform, and different concentrations of NO_2_ are tested in this section.

**Figure 1:**
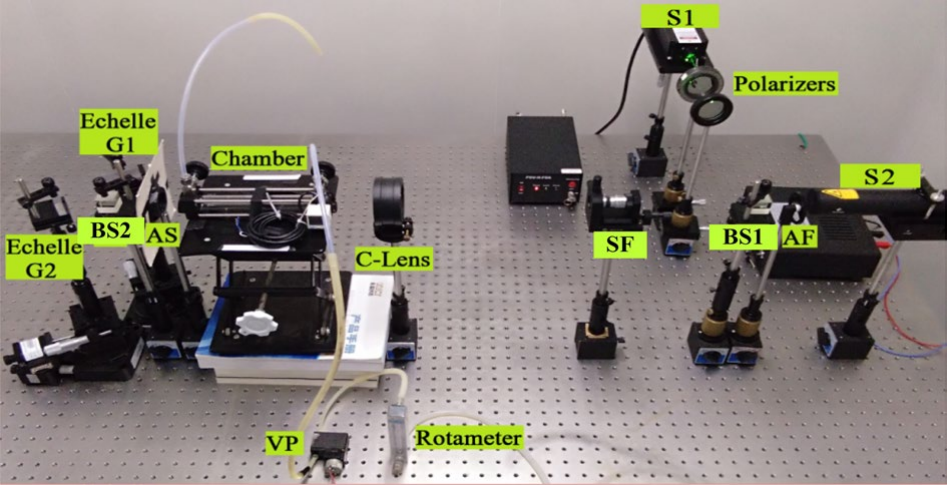
Experimental platform of visual gas sensing system with narrow band.

It should be noted that one reason why we choose the dual excitation light source is to improve the calibration accuracy of the optical path (Especially the optical path of WS-SHS), the other is to obtain the strong absorption band and weak absorption band of NO_2_ at the same time. It is a pity that there is no suitable double excitation light source in our laboratory, so a dual-laser synthesis method is used to provide a dual-wavelength input light source (shown in Fig. 1).

### Experimental platform

The experimental platform of visual gas sensing system is shown in Fig. 1.

BS indicates beam splitter, AF indicates attenuation film, SF indicates spatial filter, C-lens indicates collimating lens, AS indicates aperture slot, VP indicates vacuum pump.

In the visual gas sensing experimental platform (shown in Fig. 1), the sources S1 and S2 are used to synthesize the dual excitation light source, which not only provides energy for the sensing system, but also achieves the optical path calibration. AF is used to adjust the intensity of the input light source, BS1 is used for the synthesis of two input light source, SF and C-Lens are used to collimate the light sources, Chamber not only stores the test gas, but also serves as the place where light source and test gas react with each other. VP and Rotameter respectively control the type and the concentration of the test gas. BS2, Echelle G1 and Echelle G2 are used to build WS-SHS system, which display the response spectrum of the test gas in the form of a 2D interferogram.

Technical parameters of main devices in the experimental platform are as follows: The central wavelength of S1 is 532 nm, which is the strong absorption band of NO_2_^22^, and the output power is 100 mW. The central wavelength of S1 is 633 nm, which is the weak absorption band of NO_2_, and the output power is 2 mW. The light path of the Chamber is 200 mm and the diameter is 25 mm, the blaze angle of the Echelle gratings are 63° and the spectral range is UV-57 um. Then technical parameters of the visual gas sensing system are obtained and the spectral range is 400–700 nm, the resolution limit is 0.0355 mm^−1^.

### Test experiment

**Figure 2:**
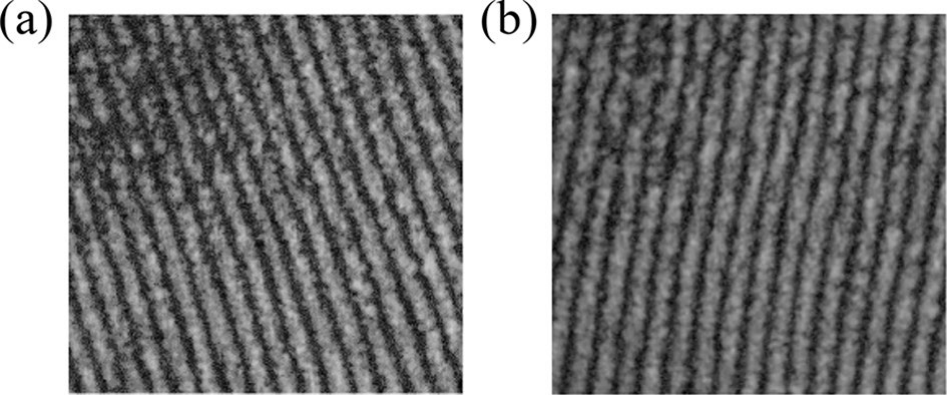
Output maps of different bands (**a**) S1, (**b**) S2.

**Figure 3:**
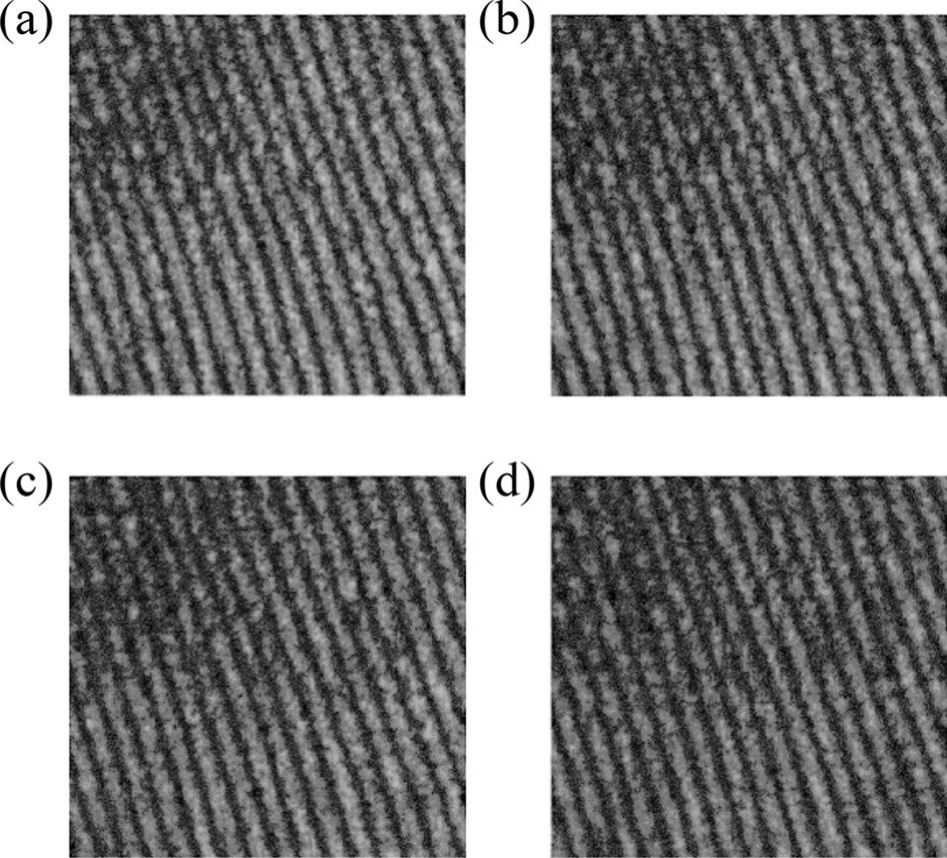
Output maps of different concentrations of NO_2_ (collected by single-band light) (**a**) N_2_, (**b**) 0.6%, (**c**) 0.8%, (**d**) 1.0%.

**Figure 4:**
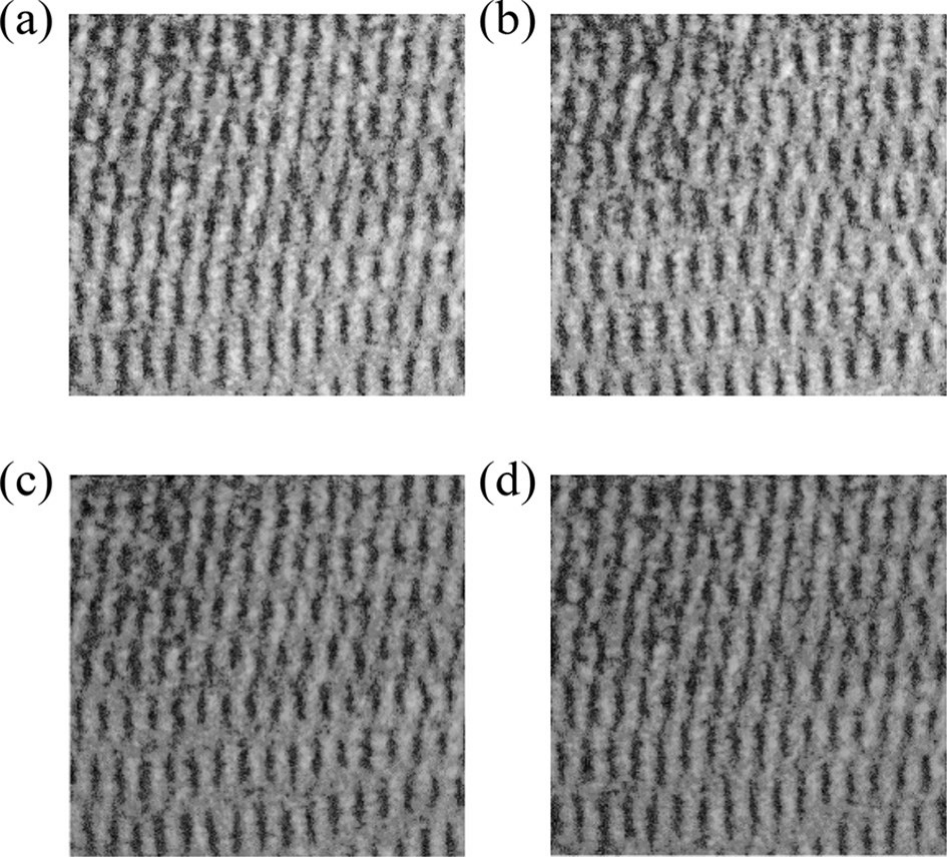
Output maps of different concentrations of NO_2_ (collected by dual-band light) (**a**) N_2_, (**b**) 0.6%, (**c**) 0.8%, (**d**) 1.0%.

In order to ensure the validity of the experimental data, this experiment was carried out in an optical darkroom with a cleanliness of 100,000 (The number of dust particles with a diameter greater than or equal to 0.5um in each cubic meter of air is less than 100,000, which indicates that the cleanliness of the laboratory is very high). The laboratory temperature is 20℃, which is basically in line with international standards^22^. And the detailed experimental process is as follows:

*Step 1:* Use N_2_ to test the air tightness of the platform and clean the Chamber and gas path;

*Step 2:* Turn on S1, then collimated light is input into WS-SHS to derive map of S1 (shown in Fig. 2a);

*Step 3:* Turn off S1 and turn on S2, then collimated light is input into WS-SHS to derive map of S2 (shown in Fig. 2b);

*Step 4:* Turn on S1, use Vacuum pump and Rotameter to charge the different concentrations of NO_2_ (different concentrations of NO_2_ are prepared with different proportions of standard NO_2_ and N_2_) into Chamber, the transmittance maps of the corresponding gas is shown in Fig. 3;

*Step 5:* Turn on S1, S2, use Vacuum pump and Rotameter to charge the different concentrations of NO_2_ into Chamber, the transmittance maps of the corresponding gas is shown in Fig. 4;

*Step 6:* At the end of the experiment, use N_2_ to clean the Chamber and gas path, then turn off the equipment.

*Step 7:* Use the SHS map correction scheme described in literature^23^, the transmittance maps are corrected respectively.

### Data analysis

#### Analysis the output interferogram of different light sources

Output interferogram of different input spectra were obtained by S1 and S2 (shown in Fig. 2). Then correlation coefficient (CC) and structure similarity coefficient (SSIM) are used to analyze the above maps.

1. Correlation coefficient

The correlation coefficient (CC) is defined as^24^:

5 $$ R = \frac{{n\sum {xy} - \sum x \sum y }}{{\sqrt {n\sum {x^{2} } - (\sum x )^{2} } \cdot \sqrt {n\sum {y^{2} } - (\sum y )^{2} } }} $$

where $x,\;y$ are variables, $n$ is the number of variables. The theoretical value of R ranges [− 1, 1]. For image processing, the range of R is [0, 1]. The closer the value is to 1, the more similar the two images are.

(2) Structure similarity coefficient

Structure similarity coefficient (SSIM) compares the similarity between images from brightness, contrast and structure. The definition is^25^,

6 $$ \begin{aligned} SSIM[f(x,y),\;g(x,y)] & = \left| {l\frac{{2\mu_{f} \mu_{g} + C_{1} }}{{\mu_{f}^{2} + \mu_{g}^{2} + C_{1} }}} \right|^{\alpha } \\ & \quad \cdot \left| {c\frac{{2\sigma_{f} \sigma_{g} + C_{2} }}{{\sigma_{f}^{2} + \sigma_{g}^{2} + C_{2} }}} \right|^{\beta } \cdot \left| {s\frac{{2\sigma_{fg} + C_{3} }}{{\sigma_{f} \sigma_{g} + C_{3} }}} \right|^{\gamma } \\ \end{aligned} $$

where $f(x,y),\;g(x,y)$ are two images to be compared, and the three items on the right are results of the comparison in brightness, contrast and structure. $\alpha ,\;\beta ,\;\gamma$ are all positive values used to adjust the importance of brightness, contrast and structure. The value of SSIM ranges [0, 1]. The greater the value is, the more similar the two images are.

Results of the output maps above evaluation parameters are shown in Table 1.

**Table 1:** CC and SSIM of output maps corresponding to different input bands.

Class	CC		SSIM	
	S1	S2	S1	S2
S1	1	0.0087	1	0.9832
S2	0.0087	1	0.9832	1

From Table 1, it can be found that: the CC of the corresponding output maps of different input spectrum is far less than 1, and the SSIM is also less than 1. It shows that for the visual gas sensing system, there are distinct differences between the output maps generated by different band spectra, which can be used as the basis for determination of gas species based on the principle of molecular spectroscopy (i.e., different gases have different absorption spectra).

#### Analysis of interferogram of single sources for different concentrations of NO_2_

The output maps of NO_2_ at different concentrations were collected in “Test experiment” section. In this section the effect of gas absorption are illustrated by sum of the gray values (SoG) and the contrast of the output map. Among them, SoG is used to represent the overall amplitude variation of the output map: the smaller SoG is, the more obvious effect of gas absorption is. Contrast is also used to characterize the absorption of the gas: the larger Contrast is, the less obvious effect of gas absorption is. Relevant parameters for gas concentration detection using S1 (shown in Fig. 3) are shown in Table 2.

**Table 2:** Parameters of NO_2_ at different concentrations (collected by single-band light).

Class	N_2_	0.6%	0.8%	1.0%
SoG	3.8 × 10^7^	3.3 × 10^7^	2.8 × 10^7^	2.5 × 10^7^
Contrast	3.858	3.6441	3.5896	3.5538

Analyzing Table 2, SoG and Contrast of the output map decrease significantly with the decrease of concentration, indicating that the test gas has a significant selective absorption to the input spectrum. The higher concentration is, the more significant absorption effect is, which is precisely subject to Lambert Beer's law (shown in Eq. (1)). More importantly, the experimental results shows that the system can be used to detect the concentration of gas. For further explanation of the gas absorption characteristics, S2 was chosen as the reference wavelength.

#### Analysis of interferogram of mixed light sources for different concentrations of NO_2_

Similarly, SoG and Contrast are used to calculated S1 and S2 mixed light source corresponding to different concentrations of NO_2_ (shown in Fig. 4). Related parameters are shown in Table 3.

**Table 3:** Parameters of NO_2_ at different concentrations (collected by dual-band light).

Class	N_2_	0.6%	0.8%	1.0%
SoG	4.7 × 10^7^	4.3 × 10^7^	4.2 × 10^7^	4.1 × 10^7^
Contrast	3.9944	4.0694	4.2841	4.4293

In Table 3, SoG of the output map is significantly reduced, but Contrast increased. According to Lambert Beer's law (shown in Eq. (1)), this is mainly due to the fact that, S1 is strongly absorbed by NO_2_, while S2 is scarcely absorbed by NO_2_. Therefore, in the dual-source output map, the Contrast of S1 decreased, while the Contrast of S2 almost did not change, resulting in an increase in the total Contrast of the output maps.

### Summary of this section

From the experimental results in “Test experiment” and “Data analysis” sections, it can be found that: Firstly, there are obvious structural differences between the output maps of different input spectra, which can be used as a basis for determining the type of gas. Secondly, the output spectral structure of NO_2_ at different concentrations is the same, while the difference is reflected in the overall amplitude of the output maps, which can be used as the detection basis of the gas concentration. To sum up, the experimental results are consistent with the theoretical derivation of V-GST, which verifies the feasibility and effectiveness of the visual gas sensing experimental platform.

## NO_2_ detection based on the new visual gas sensing experimental platform

“Experimental platform of visual gas sensing technology” section verifies the feasibility of gas sensing based on the V-GST experimental platform. This section will use a wide-spectrum light source with different central wavelength filters to detect NO_2_, and compare the difference of NO_2_ response maps in different bands to analyze the effectiveness of the visual gas detection system. The experimental methods are as follows:

### Experimental platform

**Figure 5:**
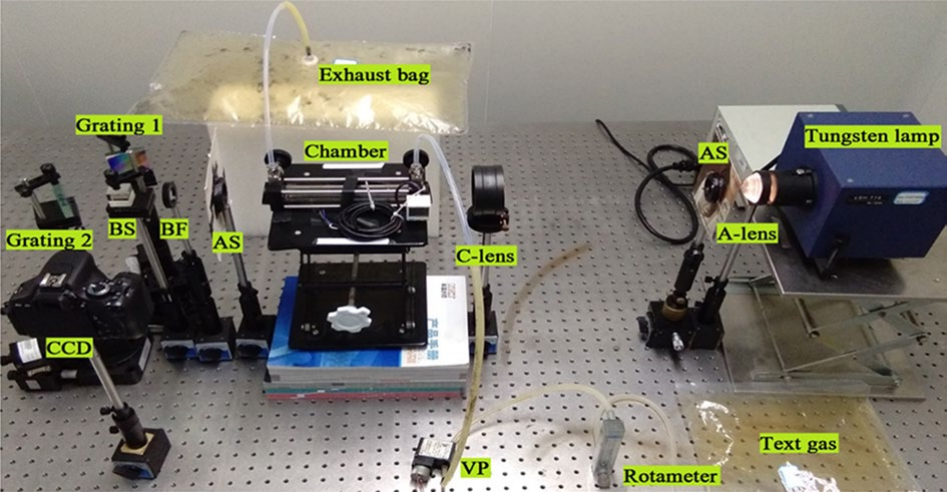
Experimental platform of visual gas detection system with wideband.

The experimental platform of NO_2_ visual gas detection system is shown in Fig. 5. And the technical parameters of main devices are as follows: BF1 has a central wavelength of 486.7 nm, which is the strong absorption band of NO_2_^22^, and the half bandwidth is 15 nm; BF2 has a central wavelength of 596.7 nm, which is the weak absorption band of NO_2,_ and the half bandwidth is 15 nm; light path of the Chamber is 200 mm and the diameter is 25 mm, the groove density of the grating is 1200 l/mm and the spectral range is 300–1000 nm. Then technical parameters of the visual gas sensing system are obtained: the resolution limit and diffraction angle of BF1 is 0.1593 mm-1 and 16.9796°, the resolution limit and diffraction angle of BF2 is 0.1299 mm-1 and 20.9795°.

A-lens indicates aspheric lens, AS indicates aperture slot, C-lens indicates collimating lens, BF indicates bandpass filter, BS indicates beam splitter, VP indicates Vacuum pump.

In Fig. 5, the Tungsten lamp not only provides energy for the sensing system, but also provides characteristic absorption lines for the test gas. A-lens is used to focus the input light, AS and C-Lens are used to collimate the light source, Chamber not only stores the test gas, but also serves as the place where light source and test gas react with each other. VP and Rotameter respectively control the type and the concentration of the test gas. BF is used for the selection of effective band. BS, Grating1 and Grating2 are used to build WS-SHS system, which displays the response map of the test gas in the form of a 2D interferogram. CCD is used to collect and store response maps.

### Experiment

#### One-dimensional test of visual gas detection system

**Figure 6:**
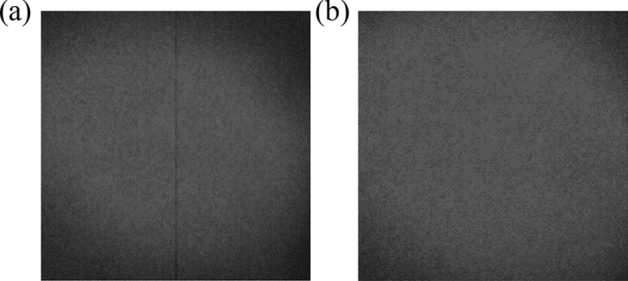
(**a**) Interferogram from BF1 of the 1D interferometer, (**b**) Interferogram from BF1 of the 2D interferometer.

It can be seen from Eqs. (2) and (3), if $\alpha = 0$, interference fringes vertical parallel to the $x$ axis, and the spatial frequency of the interference fringe along $x$ axis is $f_{x} = 4(\sigma - \sigma_{0m} )\tan {\uptheta }$^26^. Figure 6a shows the 1D interferogram under the initial alignment of each device in the visual gas sensing system, and Fig. 6b shows the 2D interferogram.

Observing Fig. 6a, it can be found that, a high-contrast line runs vertically through image center $(x = 0)$ represents the location of zero path difference, which means the gratings in the system are now adjusted to the optimal vertical state. And the inverse Fourier transform of the 1D interferogram can directly invert the spectral information of the input light, so the system was often used as a spaceborne spectrometer. But, when one or both arms of the interferometer rotate along $x$ axis, the visible lines in the interferogram disappear and become the 2D interferogram shown in Fig. 6b. In this section, the 1D interferogram of the SHS was obtained, and the effectiveness of the experimental platform was verified.

#### NO_2_ detection experiment for 2D visual gas detection system

In practical application, on the one hand, the 1D WS-SHS system will cause the overlap of the characteristic spectrum, which will affect the detection effect of the test gas. On the other hand, it is extremely difficult to adjust the optical path of the 1D WS-SHS system. Therefore, this paper chooses 2D WS-SHS as the spectral detection way of visual gas detection system.

**Figure 7:**
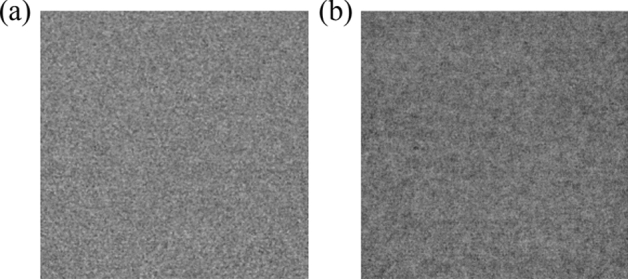
BF1 response image (**a**) System interferogram, (**b**) NO_2_ interferogram.

**Figure 8:**
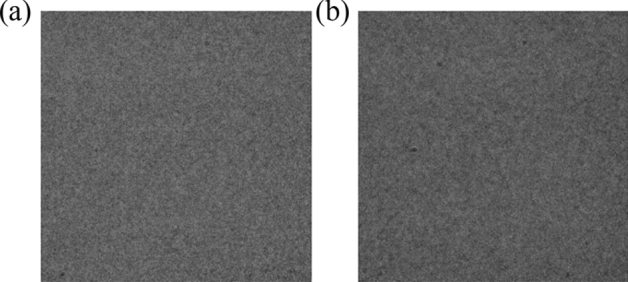
BF2 response image (**a**) System interferogram, (**b**) NO_2_ interferogram.

**Figure 9:**
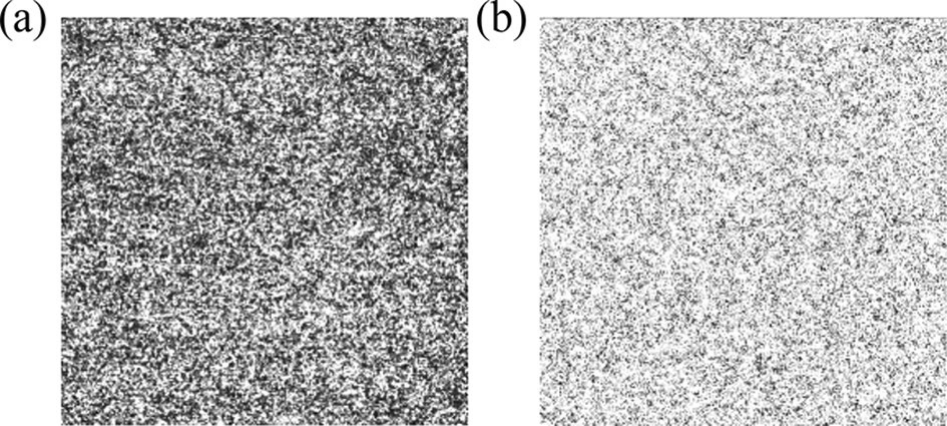
NO_2_ response image (**a**) BF1, (**b**) BF2.

This experiment was also carried out in an optical darkroom with a cleanliness of 100,000, and the laboratory temperature is 20℃. The experimental steps of NO_2_ are as follows:

*Step 1:* Use N_2_ to test the air tightness of the platform and clean the Chamber and gas path;

*Step 2:* Turn on the bromine tungsten lamp, focus and collimate the light source;

*Step 3:* The optical path is loaded with BF1 (the center wavelength is 486.7 nm), and the grating is adjusted according to the diffraction angle of 16.9796° to obtain a systematic interferogram shown in Fig. 7a;

*Step 4:* Use Vacuum pump and Rotameter to charge NO_2_ into Chamber, and the absorption interferogram of NO_2_ is obtained, as shown in Fig. 7b;

*Step 5:* Charge N_2_ into the Chamber to clean the Chamber and air path;

*Step 6:* Remove BF1 and load BF2 (the center wavelength is 596.7 nm), then adjust the grating according to the diffraction angle of 20.9795° to obtain the interferogram shown in Fig. 8a;

*Step 7:* Use Vacuum pump and Rotameter to charge NO_2_ into Chamber, and the absorption interferogram is obtained, as shown in Fig. 8b;

*Step 8:* At the end of the experiment, use N2 to clean the Chamber and gas path, then turn off the equipment;

*Step 9:* Use the SHS map correction scheme described in literature^23^, the interferograms are corrected respectively;

*Step 10:* According to the calculation scheme of the visual gas detection system, the response images of NO_2_ in BF1 and BF2 are shown in Fig. 9a,b.

Observing the response maps of NO_2_ in different bands shown in Fig. 9, it was found that the overall amplitude in Fig. 9a is lower than that of Fig. 9b, indicating that the absorption of NO_2_ in BF1 is stronger than that of BF2. According to Lambert Beer's law (shown in Eq. (1)), this result is consistent with absorption characteristics of NO_2_^22^. However, it is difficult to observe the difference between the response images of the two bands subjectively, except the overall amplitude difference of the image. It is mainly because of the wide bandwidth of the input spectrum, which seriously reduces the contrast of the response image. Therefore, the performance of the visual gas detection system will be expected to improve by effective data processing methods.

### Data analysis

Each set of experiment in “Experiment” section was repeated 80 times, and a series of images were collected. In this section, 72 images were selected randomly from the response images of NO_2_ in BF1 and BF2 to form the data set of the visual gas detection system for subsequent data analysis.

#### Feature extraction

**Figure 10:**
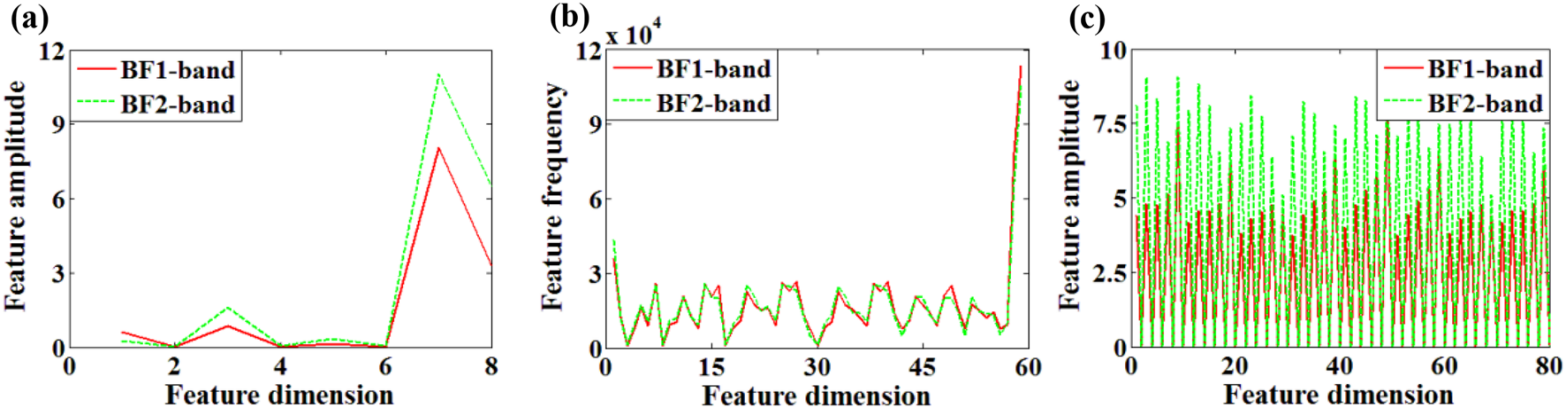
Feature extraction results of response image (**a**) GLCM feature, (**b**) LBP feature, (**c**) Gabor feature.

Analytical Eqs. (2) and (3) show that the interferogram generated by the 2D SHS system has clear texture information, so the interferogram generated by SHS can be regarded as gray-scale texture image for feature extraction. And the typical texture image feature extraction algorithms like local binary pattern (LBP)^27^, Gray-Level Co-occurrence Matrix (GLCM)^28^, Gabor wavelet transform (Gabor) are used to extract the features of the interferogram in the above data sets. The characteristic distribution of the sample is shown in Fig. 10.

Diverse algorithms have different feature information and dimensions due to different principles: Dimension of GLCM feature is 8, which represents mean and standard deviation of energy, entropy, moment of inertia and correlation of the gray level co-occurrence matrix. Dimension of LBP feature is 59, which means 59 local binary patterns. Dimension of Gabor feature is 80, which indicates the mean and standard deviation of the Gabor transform coefficients of the image.

Observing Fig. 10, it can be found that for response images of different bands, the difference of the feature distributions by GLCM and DFB is relatively obvious, while the distribution of LBP features is similar.

#### PCA analysis

**Figure 11:**
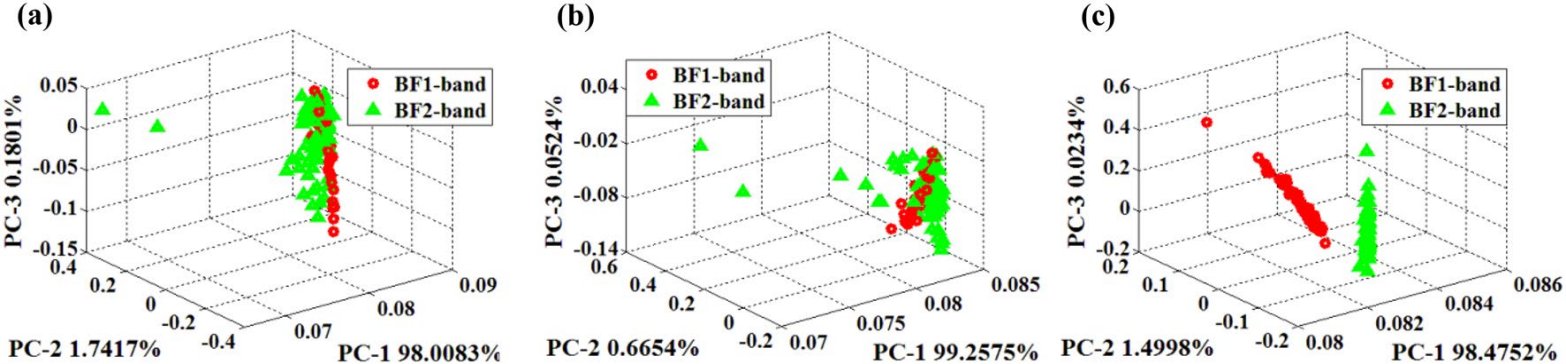
PCA scatter-gram of different feature extraction algorithms (**a**) GLCM-PCA, (**b**) LBP-PCA, (**c**) Gabor-PCA.

In order to reduce the dimension of the feature data of the test gas and display the difference of response data more intuitively, this paper uses PCA^29^ to reduce the dimension of the feature data in “Feature extraction” section  (shown in Fig. 10). The scatter-grams of PCA analysis are as follows:

In Fig. 11, PC1, PC2, PC3 respectively represent the percentages of the original information contained in each principal component of PCA analysis, while the signs in color red and green represent samples in different bands. The cumulative contribution rate of the first three principal components exceeds 99.9%, indicating that the first three principal components of the PCA analysis already contain most of the information of the original data. However, in Fig. 11a,b, the PCA scatter distribution of GLCM and LBP features overlaps seriously. In Fig. 11c, the PCA scatter distribution of Gabor features shows that the same kind of samples are gathered while different kind of samples are relatively separated, which improves the probability of distinguishing the two kind of samples and shows the superiority of the proposed algorithm.

#### Type recognition of the experiment sensing data

After feature extraction and PCA dimension reduction of the original sample data, the first three principal components vector of PCA contains the main information of the test gas was selected to form a new Dataset. According to the proportion of 7: 3, the Kennard-Stone sequential (KSS) algorithm^30^ was used to allocate the new sample set to the training set and the test set and classification analysis was performed at last.

The typical classification algorithms like correlation coefficient (CC)^31^ and Euclidean distance to centroids (EDC)^32^ were used to pattern recognition of the new Dataset. And the classification accuracy is shown in Table 4, it can be found: ① the mean classification accuracy of the Dataset reaches more than 80%, which shows that the visual gas detection system constructed in this paper can reflect the information of the test gas well; ② The overall recognition rate of CC algorithm is higher than that of EDC algorithm; ③ The classification accuracy of Gabor algorithm is higher than that of GLCM and LBP, and the classification accuracy reaches more than 95%, which shows that Gabor feature extraction is the best algorithm for this system.

**Table 4:** Classification accuracy of the new Dataset.

Class	CC			EDC		
	GLCM	LBP	Gabor	GLCM	LBP	Gabor
BF1-band accuracy	75	100	100	75	91.67	100
BF2-band accuracy	70.83	83.33	95.83	41.67	41.67	95.83
Mean	72.92	91.66	97.92	58.33	66.67	97.92

## Conclusion

In this paper, in order to verify the effectiveness of the V-GST, the theoretical model of V-GST was constructed, then a V-GST experimental platform was built, and the visual transmittance maps of NO_2_ with different concentrations and different spectra were collected, thirdly the typical feature extract (such as LBP, GLCM, Gabor), PCA and classification algorithms (such as CC, EDC) were chosen to process the response data. Results show that: V-GST not only has different interferogram response to different spectra, but also has a good response to different concentrations of gas, which lays a foundation for the application of this technology in gas detection. Then, using the texture image feature extraction algorithm can greatly reduce the amount of data of the original response data and improve the processing efficiency of the data. Finally, the average classification recognition rate of the system for different band of NO_2_ response data is over 80%, which verifies the effectiveness of the visual gas sensing system in gas detection.

Of course, there are still some deficiencies in the current research, such as the type of test gas is relatively single, the limit detection concentration of NO_2_ is relatively high. That is to say, the performance indicators of the system are far from meeting the requirements of daily ambient gas detection. So, in order to expand the application field and application prospect of the V-GST system, these shortcomings will be improved in the follow-up research.
